# Myeloid IκBα Deficiency Promotes Atherogenesis by Enhancing Leukocyte Recruitment to the Plaques

**DOI:** 10.1371/journal.pone.0022327

**Published:** 2011-07-21

**Authors:** Pieter Goossens, Monique N. Vergouwe, Marion J. J. Gijbels, Danielle M. J. Curfs, Johannes H. G. van Woezik, Marten A. Hoeksema, Sofia Xanthoulea, Pieter J. M. Leenen, Rudolf A. Rupec, Marten H. Hofker, Menno P. J. de Winther

**Affiliations:** 1 Department of Molecular Genetics, Cardiovascular Research Institute Maastricht, Maastricht University, Maastricht, The Netherlands; 2 Department of Pathology, Cardiovascular Research Institute Maastricht, Maastricht University, Maastricht, The Netherlands; 3 Department of Immunology, Erasmus University Medical Center, Rotterdam, The Netherlands; 4 Department of Dermatology, Ludwig-Maximilian-University, Munich, Germany; 5 Department of Pathology and Medical Biology, Medical Biology Section, Molecular Genetics, University Medical Center Groningen, University of Groningen, Groningen, The Netherlands; University of Cambridge, United Kingdom

## Abstract

Activation of the transcription factor NF-κB appears to be involved in different stages of atherogenesis. In this paper we investigate the role of NF-κB inhibitor IκBα in atherosclerosis. Myeloid-specific deletion of IκBα results in larger and more advanced lesions in LDL-R-deficient mice without affecting the compositional phenotype of the plaques or systemic inflammatory markers in the plasma. We show that IκBα-deleted macrophages display enhanced adhesion to an in vitro endothelial cell layer, coinciding with an increased expression of the chemokine CCL5. Also, in vivo we found that IκBα^del^ mice had more leukocytes adhering to the luminal side of the endothelial cell layers that cover the atherosclerotic plaques. Moreover, we introduce ER-MP58 in this paper as a new immunohistochemical tool for quantifying newly recruited myeloid cells in the atherosclerotic lesion. This staining confirms that in IκBα^del^ mice more leukocytes are attracted to the plaques. In conclusion, we show that IκBα deletion in myeloid cells promotes atherogenesis, probably through an induced leukocyte recruitment to plaques.

## Introduction

NF-κB is a transcription factor that translates the inflammatory stimuli from the environment into gene expression patterns regulating cell differentiation, activation, proliferation and apoptosis as well as the production of a set of inflammatory mediators. It is activated in response to pathogen detection by Toll-like receptor signaling or, in the inflammatory milieu, through different cytokine receptors such as the TNF receptor. Also, non-pathogen related activation, called “sterile inflammation”, including stimuli such as free radicals, radiation and modified lipoproteins, can trigger NF-κB [1], [2].

Rather than one transcription factor, NF-κB is in fact a family of homo- and heterodimers, with different possible combinations of the Rel-domain containing proteins NF-κB1 (p50 and its precursor p105), NF-κB2 (p52 and its precursor p100), RelA (p65), RelB and c-Rel. In the absence of an activating stimulus, the NF-κB dimer is kept cytoplasmic because its nuclear localization signal is covered by an inhibitor belonging to the IκB family [3]. The IκB family consists of IκBα, IκBβ, IκBγ, IκBε and Bcl-3 [4]. Following a signaling cascade initiated by an inflammatory stimulus, IκB is phosphorylated by a complex consisting of IκB kinase 1 (IKK1 or IKKα), IKK2 (or IKKβ) and NEMO (or IKKγ). This phosphorylation leads to ubiquitination and subsequent proteasomal degradation of the IκB, leaving NF-κB free to translocate to the nucleus [5]. Being NF-κB target genes themselves, the IκB family members are part of a negative feedback loop, retracting NF-κB from the nucleus back into the cytoplasm and thereby preventing excessive and irreversible NF-κB activation [6].

NF-κB activation is an important response in different infectious as well as non-infectious pathologies. Also in the different stages of atherogenesis, from early endothelial activation to eventual plaque rupture, NF-κB has been described as a key regulator [7], [8]. Atherosclerosis is a slowly progressing, chronic inflammatory disease of the large arteries representing the most common cause of death in western society [9]. This process is initiated when modified lipoproteins in the vessel wall activate the endothelial lining of the vessel, thereby attracting monocytes, which differentiate into macrophages upon migration through the endothelium. By taking up and storing the lipoproteins, these macrophages eventually become large foam cells and start secreting inflammatory mediators, cytokines and chemokines. The thereby created inflammatory environment attracts even more monocytes as well as other immune cells to the vessel wall, forming an atherosclerotic plaque [10].

In the atherosclerotic plaque, a wide variety of NF-κB inducers is present, ranging from modified lipoproteins to inflammatory mediators, free radicals and remnants of dead cells [11], [12], [13]. Therefore, activated NF-κB has been found in different cell types in the lesion, including macrophages, smooth muscle cells and endothelial cells [8], [14]. To investigate the importance of this activation, we have previously studied models with either a macrophage- [15] or endothelial cell-specific [16] ablation in NF-κB activation. While macrophage-specific deletion of IKK2 led to larger and more advanced lesions, endothelium-restricted NEMO deletion abrogated atherogenesis by impairing macrophage recruitment to the plaque.

In this paper we aimed at investigating the role of the NF-κB inhibitor IκBα in atherogenesis. Since full IκBα knockout mice die neonatal of hypergranulopoiesis and severe dermatitis [17], [18], [19], we used a conditional model with a myeloid specific deletion of IκBα [20]. Bone marrow from these LysMCre-*IκBα* ^fl/fl^ mice was transplanted into atherosclerosis-susceptible *ldlr* ^−/−^ mice to study the effect of myeloid IκBα deficiency on atherogenesis. We found that myeloid IκBα deficiency promotes atherogenesis by causing increased attraction of myeloid cells to the developing plaques without affecting other phenotypical characteristics of the lesions. Quite surprisingly, macrophage IκBα deficiency does not seem to affect the production of a number of NF-κB target genes *in vivo* nor *in vitro* but appears to be involved in the adhesion and recruitment of these cells to the atherosclerotic plaque.

## Results

### Myeloid IκBα deficiency promotes atherogenesis without altering plaque phenotype, body weight, plasma lipids and cytokines

**Figure 1:**
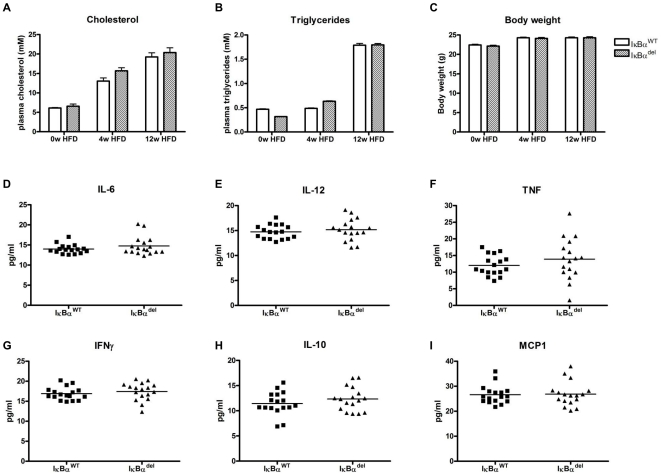
Plasma values and body weight of mice transplanted with either *IκBα* ^fl/fl^ or LysMCre-*IκBα* ^fl/fl^ bone marrow. (A) Cholesterol levels, (B) triglyceride levels and (C) body weight measured before the start of the high fat diet (0w HFD), after 4 weeks of diet (4w HFD) and upon sacrifice of the mice (12w HFD). (D–I) Plasma cytokine concentrations at the end of the experiment, measured by a bead array.

To study the effect of myeloid IκBα deficiency on atherosclerosis, bone marrow was isolated from *IκBα* ^fl/fl^ and LysMCre-*IκBα* ^fl/fl^ mice, the latter having a myeloid-specific deletion of the *IκBα* gene [20]. This bone marrow was transplanted into irradiated *ldlr* ^−/−^ mice, resulting in atherosclerosis-susceptible mice that were either wildtype (IκBα^WT^) or deleted (IκBα^del^) for *IκBα* in their myeloid cells. Four weeks after transplantation, these mice were put on a high fat diet for 12 weeks in order to induce atherogenesis. The rise in plasma cholesterol, plasma triglycerides and body weight as a result of the diet was similar in both groups (Figures 1A, 1B and 1C). In addition, relative levels of circulating leukocyte populations (monocytes, granulocytes, T and B lymphocytes) after the transplantation did not differ between the groups (data not shown). After 12 weeks of diet, plasma levels of the NF-κB dependent pro-inflammatory cytokines IL-6, IL-12, TNF-α and IFNγ, the anti-inflammatory cytokine IL-10 and the chemokine MCP-1 (CCL2) were not influenced by the absence of myeloid IκBα (Figures 1D–1I).

**Figure 2:**
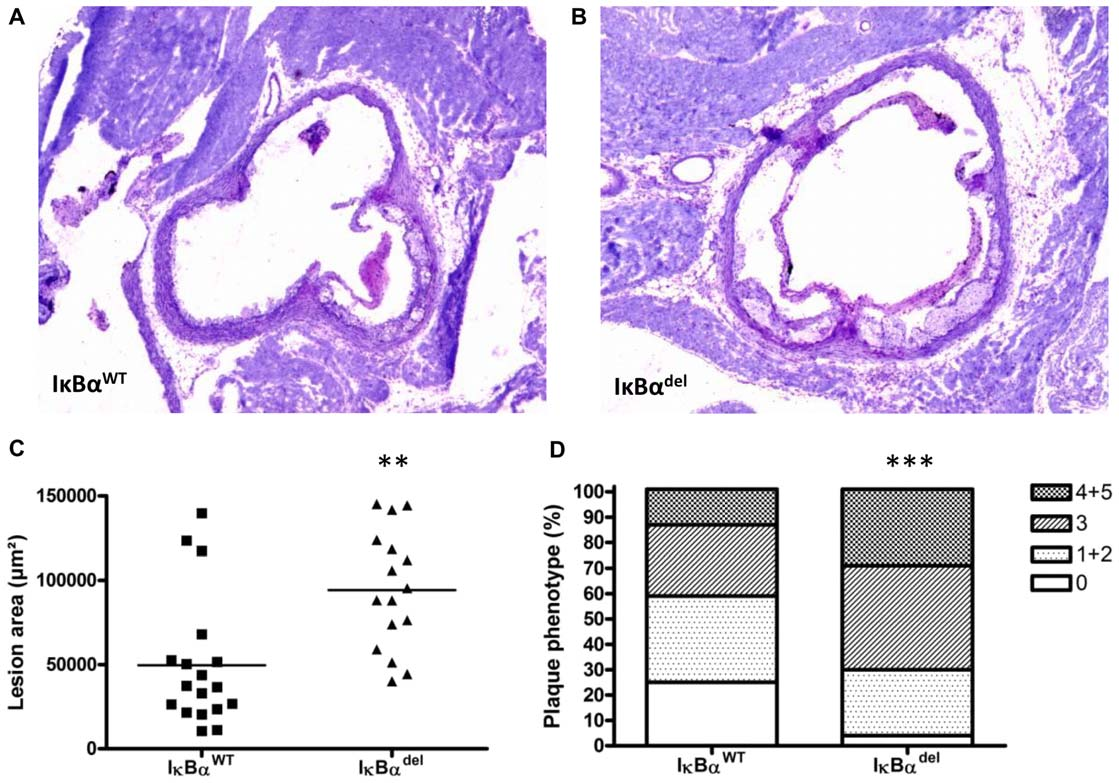
Myeloid IκBα deficiency promotes atherosclerosis in *ldlr* ^−/−^ mice. Representative pictures of toluidine blue-stained sections in the aortic root of (A) IκBα^WT^ or (B) myeloid-specific IκBα^del^ mice, original magnification ×40. (C) Lesion area in the aortic root of IκBα^WT^ and IκBα^del^ mice (** p<0.01; n = 18/16). (D) Lesion severity in the aortic root of IκBα^WT^ and IκBα^del^ mice (Chi square test; *** p<0.0001; n = 54/48) was typed as absent (0), early (1+2), or advanced (4+5), as described before [15].

Upon sacrifice, atherosclerosis in the aortic root was analyzed. Interestingly, lesion area measurements using toluidine blue-stained sections (Figures 2A, 2B) showed a two-fold, significant increase in plaque formation in IκBα^del^ mice compared to IκBα^WT^ mice (49740±9142 µm^2^ vs. 94330±8803 µm^2^ for IκBα^WT^ and myeloid-specific IκBα^del^ respectively; p = 0.0014) (Figure 2C). Moreover, classification of the lesions according to their severity showed that IκBα^del^ mice had relatively more advanced and less early atherosclerotic lesions (Chi square test; p<0.0001) (Figure 2D).

**Figure 3:**
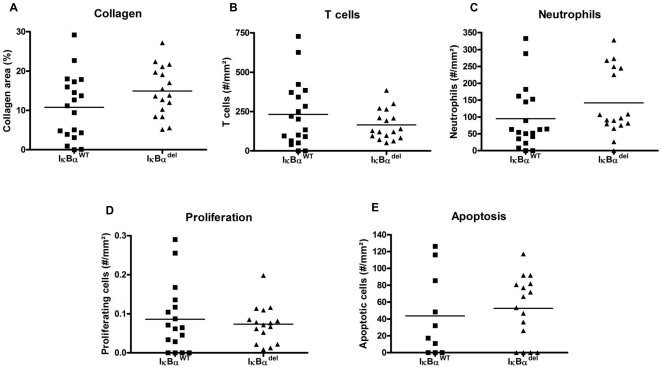
Myeloid IκBα deficiency does not influence plaque phenotypic characteristics. (A) Collagen content is not different between IκBα^WT^ and IκBα^del^ mice, as shown by a Sirius Red staining. Also the relative number of (B) T cells, (C) neutrophils, (D) proliferating and (E) apoptotic cells was similar in both groups.

With additional analyses on sections of the aortic root, the phenotype of these plaques was further characterized. Plaque-stabilizing collagen was stained with Sirius Red but no significant differences were found in collagen content between the two groups (Figure 3A). Also analysis of T cell and neutrophil content revealed no significant differences (Figures 3B and 3C). Finally, both groups appeared to have the same number of proliferating and apoptotic cells in their plaques, as shown by Ki-67 and a TUNEL stainings (Figures 3D and 3E). Thus despite having larger and more advanced lesions, the IκBα^del^ lesions showed no other changes in their plaque characteristics.

Next to the lesions in the aortic root, atherosclerotic plaque formation was also assessed in the aortic arch. In RNA isolated from this tissue, a 49.6% increase in the expression of macrophage marker CD68 was found (p = 0.0384, data not shown), indicating enhanced macrophage accumulation in the vessel wall of IκBα^del^-transplanted mice.

### Myeloid IκBα deficiency promotes in vitro macrophage adhesion

**Figure 4:**
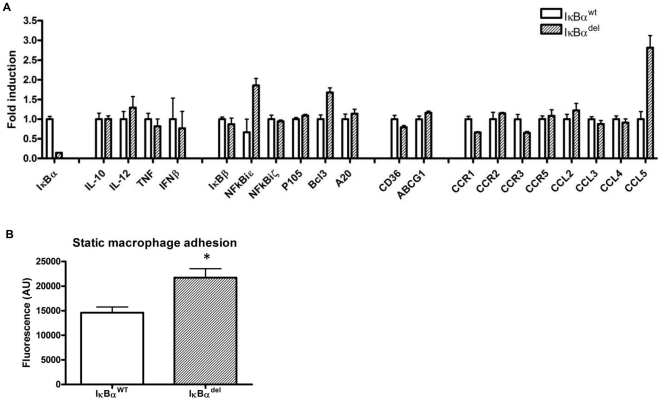
Deletion of *IκBα* in bone marrow-derived macrophages has limited effect on the expression of NF-κB-dependent and/or –regulating genes but enhances *in vitro* static macrophage adhesion. (A) Gene expression in triplicates of LysMCre-*IκBα* ^fl/fl^ macrophages was compared to *IκBα* ^fl/fl^ macrophages by Q-PCR. (B) Adhesion of fluorescently labeled macrophages to an endothelial monolayer (bars represent triplicate wells ± SEM; * p<0.05). Data shown are representative for at least 3 experiments.

To investigate the mechanism behind the enhanced atherogenesis in the IκBα^del^ mice, *in vitro* experiments were done. Surprisingly, the reduced production of IκBα shown in Figure 4A and Figure S1A did not result in an increased activation of the p65 NF-κB subunit (Figure S1B–C), suggesting a potent compensatory mechanism operating in the absence of IκBα. As a result, hardly any difference was found in the expression of some genes known to be NF-κB targets, NF-κB inhibitors or inflammatory mediators when assessed in unstimulated as well as LPS stimulated bone marrow-derived macrophages from LysMCre-*IκBα* ^fl/fl^ mice and compared to wild type cells (Figure 4A and Figure S1D). However, the expression of the chemokine CCL5 (or RANTES) was significantly elevated 2.8 fold in macrophages lacking IκBα. CCL5 was previously shown to be involved in the adhesion of macrophages to endothelial cells. In line with these findings, static adhesion of IκBα^del^ macrophages to a monolayer of the bEND.5 endothelial cell line was significantly enhanced compared to wild-type macrophages (14610±1163 AU vs. 21720±1810 AU for IκBα^WT^ and IκBα^del^ respectively; p = 0.0298) (Figure 4B).

### Atherosclerotic lesions in mice lacking myeloid IκBα show increased leukocyte adhesion and migration

**Figure 5:**
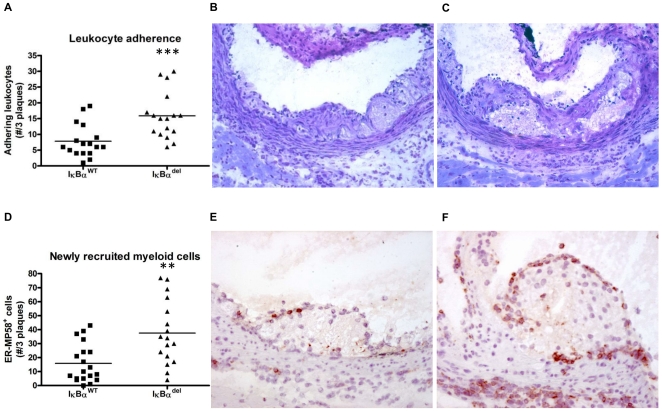
Increased leukocyte adhesion and newly recruited myeloid cells in IκBα^del^ mice. (A) In IκBα^del^ mice, more leukocytes adhere to the endothelial cell layer delineating the lesion (*** p<0.0001; n = 17/17). Representative pictures from (B) IκBα^WT^ and (C) IκBα^del^ lesions, original magnification ×200. (D) Lesions from IκBα^del^ mice contain more ER-MP58 positive cells, suggesting an increased attraction myeloid cells (** p<0.001; n = 19/17). Representative pictures from (E) IκBα^WT^ and (F) IκBα^del^ lesions, original magnification ×200.

To investigate whether the observed increased *in vitro* macrophage adhesion could also be observed in the *in vivo* atherosclerosis model, cells adhering to luminal side of the endothelial cells covering the atherosclerotic plaques were quantified in the toluidine blue-stained sections. Indeed, the IκBα^del^ mice displayed a significantly higher number of adhering cells compared to the IκBα^WT^ mice (7.8±1.3 cells vs. 15.9±1.8 cells for IκBα^WT^ and IκBα^del^ respectively; p = 0.0009) (Figures 5A–5C), suggesting enhanced recruitment of new cells to the plaque in mice lacking IκBα in their myeloid cells.

To further analyze this recruitment of monocytes to the lesions, we used a marker that is specifically expressed on circulating immature myeloid cells but that is lost upon differentiation to macrophages. This marker, detected by the antibody ER-MP58, has previously been used in other studies to analyze recruitment of cells from the circulation to tissues [21], [22], [23]. Sections from the aortic root were stained with ER-MP58 and positive cells were quantified. As expected, these cells were predominantly observed on the luminal side of the plaque. However, also on the adventitial side, some myeloid recruitment could be observed. Interestingly, ER-MP58-positive cells were also seen in inflammatory regions in the vicinity of the plaques. Quantification showed that IκBα^del^ mice had significantly more newly recruited myeloid cells in their plaques compared to IκBα^WT^ mice (15.890±3.164 cells vs. 37.590±5.568 cells; p = 0.0014) (Figures 5D–5F). These data indicate that atherosclerotic lesions in the aorta of *ldlr* ^−/−^ mice lacking myeloid IκBα may be larger because of enhanced recruitment and infiltration of myeloid cells, suggesting that myeloid IκBα is important in regulating the migratory phenotype of cells in atherogenesis. A possible role for CCL5 in this mechanism is illustrated by the observation that the expression of this chemokine was significantly elevated in the aforementioned aortic arch-derived RNA (40.7% increase, p = 0.0270, data not shown).

## Discussion

In this paper we show that myeloid-specific deletion of the inhibitor of NF-κB, IκBα, in *ldlr^−^* ^/−^ mice results in larger atherosclerotic plaques without affecting the general plaque composition. Studying cell adhesion to the luminal side of the endothelial layer covering atherosclerotic plaques, we observed increased adhesion of leukocytes in the absence of myeloid IκBα. Through a marker specific for circulating myelomonocytic cells that is lost upon maturation to macrophages, ER-MP58, we found that lesions from mice lacking myeloid IκBα are characterized by more newly recruited leukocytes. Moreover, we found an increase in macrophage content in the vessel wall of the aorta. These data suggest that IκBα in myeloid cells may have a pivotal role in regulating the recruitment of cells to atherosclerotic lesions.

Atherosclerosis is known to be driven by inflammation and aggravated by the production of pro-inflammatory cytokines [24]. Since cytokine expression is highly dependent on NF-κB, it can be expected that the atherosclerotic process is proportional to the activation level of this transcription factor. Indeed, Gareus et al. showed that endothelial-specific inhibition of NF-κB by NEMO deletion impaired macrophage recruitment to the plaque and hereby impaired atherogenesis [16]. Another paper, by Wolfrum et al., describes how haploinsufficiency for the NF-κB activation inhibitor A20 results in enhanced atherogenesis while A20 overexpression reduced plaque formation [25]. Our group also showed that the role of NF-κB in macrophages is not as straightforward. Myeloid-specific blocking of the canonical activation of NF-κB through deletion of IKK2 resulted in plaques that were not only larger but also more advanced and more necrotic, highlighting the fact that NF-κB also acts as an anti-apoptotic transcription factor and is involved in regulating anti-inflammatory mechanisms [15]. In contrast, the present study demonstrates that deletion of myeloid IκBα, aiming at myeloid-specific NF-κB activation, also induces larger and more advanced plaques but without affecting the plaque composition.

Studying gene expression in LysMCre-*IκBα* ^fl/fl^ bone marrow-derived macrophages and comparing it to wildtype cells, we found an increase in the expression of the chemokine RANTES (Released upon Activation, Normal T-cell Expressed and Secreted, or CCL5). This molecule belongs to a relatively limited set of chemokines and adhesion molecules that are described to be influencing atherogenesis through the recruitment of new cells to the plaque [26], [27]. In a recent publication, our group showed that IFNβ-induced expression of CCL5 augments the static adhesion of macrophages to endothelial cells *in vitro* as well as the adhesion of leukocytes to the vessel wall at atherosclerosis-prone sites *in vivo*, thereby promoting the attraction of new cells to the plaques [28]. Macrophages from LysMCre-*IκBα* ^fl/fl^ mice indeed adhered more efficiently to an endothelial cell layer *in vitro*, while in the atherosclerosis model, more monocytes were found adhering to the endothelial layer covering the plaques in the IκBα^del^ mice compared to the IκBα^WT^ mice.

To confirm the hypothesis that the lesions in the IκBα^del^ mice were larger because of an increased attraction of leukocytes, we applied a staining detecting a marker specific for immature myeloid cells, ER-MP58. The target is an antigen with a yet unknown function, which is present on the bone marrow-derived, myeloid-committed progenitor cells. It continues to be expressed on neutrophils and monocytes but disappears progressively upon maturation of the M-CSF-responsive cells to macrophages [21], [29]. Thus, both Ly-6C-high and Ly-6C-low subsets of circulating monocytes are positive for ER-MP58 while tissue macrophages have lost the marker [29], [30]. Previously, detection of this marker has been used to distinguish infiltrating immature myeloid cells from the resident macrophages already present within the site of inflammation, both in thioglycollate elicited macrophage recruitment to the peritoneum [21], [22] and in the restoration of the Kupffer cell population in the liver, following injection with liposome-entrapped dichloromethylene diphosphonate [23]. In this paper, we show that ER-MP58 is also a valid marker for newly recruited myeloid cells to the atherosclerotic plaque, with a positivity that is limited to small, recently infiltrated cells in the vicinity of the luminal plaque surface and inflammatory regions.

Contrary to the upregulation of CCL5 expression in the LysMCre-*IκBα* ^fl/fl^ macrophages, many typical NF-κB-dependent genes were not influenced by the deletion of IκBα. This suggests that in these cells, NF-κB is not continuously activated, but inhibited by other feedback mechanisms which, like IκBα, prevent NF-κB translocation to the nucleus or terminate NF-κB activation by exporting it back to the cytoplasm. Indeed, studying the nuclear translocation of several NF-κB subunits, we found that the LysMCre-*IκBα* ^fl/fl^ macrophages had the same degree of translocation as wildtype cells (data not shown) and no increase in p65 phosphorylation was observed in the IκBα^del^ macrophages (Figure S1B–C). In addition, only mild to no upregulation of other IκB family members was detected by gene expression analysis, indicating alternative mechanisms of regulation of NF-κB dependent transcription. Recent studies indeed show that, besides IκB inhibitors, also nuclear ubiquitin ligases can terminate chronic NF-κB activation by its nuclear degradation, a mechanism that might compensate the deletion of IκBα [31].

In conclusion, we found that myeloid-specific deletion of IκBα resulted in an upregulation of the chemokine CCL5 and enhanced static *in vitro* adhesion of macrophages to an endothelial cell layer. Moreover, this correlated *in vivo* with increased leukocyte adhesion to the activated endothelial lining of the blood vessel, enhanced recruitment of ER-MP58^+^ immature myeloid cells to the atherosclerotic plaque and increased atherosclerotic lesion formation in the aortic root and arch. Hereby we show that the role of myeloid IκBα in the regulation of inflammation is complex but that it is involved in the recruitment of macrophages to the atherosclerotic plaque.

## Materials and Methods

### Mice

C57BL/6 mice and *ldlr* ^−/−^ mice on a C57BL/6 background were obtained from Jackson Laboratory (Bar Harbor, ME). *IκBα* ^fl/fl^ mice on a C57BL/6 background were described before [32]. All animal experiments were approved by the DierExperimenten Commissie (DEC) of the Maastricht University (permit numbers 2005-090 and 2009-168).

### Bone marrow transplantation

One week before transplantation, female *ldlr* ^−/−^ mice were housed in filter top cages and provided with acidified water containing neomycin (100 mg/l; Gibco, Breda, The Netherlands) and polymyxin B sulphate (6×10^4^ U/l; Gibco). The animals received 10 Gy total body irradiation and on the following day, bone marrow was isolated from 6 LysMCre-IκBα^fl/fl^ mice (IκBα^del^) and 6 IκBα^fl/fl^ littermates (IκBα^wt^) and 10^7^ cells/mouse were injected intravenously to rescue the hematopoietic system of the irradiated mice. Four weeks after the transplantation, mice were fed a high fat diet (0.15% cholesterol, 16% fat, Arie Blok, The Netherlands) for 12 weeks.

### Mouse blood parameters

At several time points during the *in vivo* atherosclerosis experiment, blood was drawn from the mice. Plasma lipid levels were monitored enzymatically (Sigma Aldrich, Zwijndrecht, the Netherlands) and plasma cytokine levels were measured by flow cytometry using a Cytometric Bead Array kit (BD-Pharmingen, San Diego, CA). Leukocytes were counted using a Coulter counter and blood cell distribution was quantified by flow cytometry after antibody staining with either Mac1-PE and Gr1-FITC for macrophages and granulocytes or 6B2-PE and KT3-FITC for B- and T-cells (BD-Pharmingen, Erembodegem, Belgium).

### Atherosclerosis analysis

Upon sacrifice, the hearts from the bone marrow transplanted mice were taken out and cut perpendicular to the heart axis just below the atrial tips. Tissue was frozen in tissue-tec (Shandon, Veldhoven, The Netherlands) and cut into sections of 7 µm as described before [15]. Serial cross-sections from every 42 µm were stained with toluidine blue. All lesion areas were quantified using Adobe Photoshop software. The lesions were also typed according to severity as early, moderate and advanced, as described before [15].

### Immunohistochemical staining

Lesions from the aortic root were fixed in acetone and incubated with antibodies against neutrophils (1A8, BD-Pharmingen), T cells (KT3, directed against CD3, a gift from G. Kraal), proliferating cells (Ki-67, Dako, Glostrup, Denmark) and newly recruited macrophages (ER-MP58, P. Leenen), followed by detection with a biotin labeled rabbit anti-rat antibody and staining with the ABC kit (Vector Labs, Burlingame, CA). Apoptotic cells in the plaques were stained by the TUNEL staining (Roche Diagnostics, Mannheim, Germany) according to the manufacturer's protocol. Collagen areas were analyzed on Sirius red stained sections. Adhering leukocytes were quantified on toluidine blue-stained sections.

### In vitro murine bone marrow macrophage culture

Bone marrow cells were isolated from femurs and tibiae of either wild-type (IκBα^fl/fl^) or deleted (LysMCre-IκBα^fl/fl^) mice. Cells were cultured in RPMI-1640 (GIBCO Invitrogen, Breda, The Netherlands) with 10% heat-inactivated fetal calf serum (Bodinco B.V., Alkmaar, The Netherlands), penicillin (100 U/ml), streptomycin (100 ug/ml), and L-glutamine 2 mM (all GIBCO Invitrogen, Breda, The Netherlands) supplemented with 15% L929-conditioned medium (LCM) for 8–9 days to generate bone marrow-derived macrophages (BMM), as described previously [15].

### Western blotting

Protein was isolated from BMM with an SDS lysis buffer, supplemented with complete protease inhibitor cocktail (Roche Diagnostics) and PhosSTOP phosphatase inhibitor cocktail (Roche Diagnostics). After Western blotting, blots were incubated with P-p65 antibody (1∶500, Cell Signaling, Danvers, MA) in PBS with 0.05% Tween and 5% BSA (Sigma Aldrich).

### Gene expression

RNA was isolated from BMM with the High Pure RNA Isolation Kit (Roche, Basel, Switzerland) or from snap-frozen aortic arches with the RNeasy Mini Kit (Qiagen, Venlo, The Netherlands). 500 ng total RNA was reverse transcribed using the iScript cDNA Synthesis Kit (BioRad, Veenendaal, The Netherlands). Quantitative PCR (Q-PCR) was performed using 10 ng cDNA, 300 nM of each primer, and SensiMix (Quantace-Bioline, London, UK) in a total volume of 20 µl. All gene expression levels were corrected for cyclophilin A as housekeeping gene. Primer sequences are available upon request.

### In vitro adhesion assay

A confluent monolayer of bEND5 endothelial cells was grown in fluorescence 96-well microplates (Greiner Bio-one, Frickenhausen, Germany). Triplicate wells were incubated for 30 min with 10^5^ BMM that had been fluorescently labeled with a PKH67 dye according to the manufacturer's instructions (Sigma Aldrich, Zwijndrecht, The Netherlands). Subsequently, the wells were washed three times with the aforementioned macrophage medium, and adherent cells were measured by fluorometry in a Synergy HT microtiter plate reader (BioTek, Bad Friedrichshall, Germany) at an excitation of 485 nm and an emission of 520 nm.

### Statistical analysis

The statistical analyses were performed using Graphpad Prism (Graphpad Software). Differences between 2 groups were evaluated using a t-test, unless stated otherwise. Values are represented as mean ± SEM. A *P* value of less than .05 was considered to be statistically significant. All mouse data passed a normality test.

## Supporting Information

Figure S1 **Deficiency of IκBα does not lead te increased NF-κB activity and subsequent gene expression.** (A) Western blotting for IκBα on lysates of bone marrow derived IκBα^WT^ or IκBα^del^ macrophages confirmed a reduction in IκBα production, both before and after LPS activation. (B–C) A western blot for phosphorylated NF-κB subunit p65 showed that NF-κB is not continuously activated in IκBα^del^ macrophages, (D) resulting in a lack of differential expression patterns for NF-κB dependent genes.
(TIF)
